# Chloroquine and Hydroxychloroquine Myopathy: Clinical Spectrum and Treatment Outcomes

**DOI:** 10.3389/fneur.2020.616075

**Published:** 2021-02-02

**Authors:** Elie Naddaf, Pritikanta Paul, Omar F. AbouEzzeddine

**Affiliations:** ^1^Division of Neuromuscular Medicine, Department of Neurology, Mayo Clinic, Rochester, MN, United States; ^2^Department of Cardiovascular Medicine, Mayo Clinic, Rochester, MN, United States

**Keywords:** chloroquine myopathy, hydroxychloroquine myopathy, toxic myopathy, vacuolar myopathy, lysosomal myopathy

## Abstract

Chloroquine (CQ) and hydroxychloroquine (HCQ) have been associated with muscle toxicity, mostly described as a proximal myopathy with evidence of lysosomal dysfunction on muscle biopsy. In this retrospective study, we aimed to define the clinical phenotype, laboratory features, and treatment outcomes of CQ/HCQ myopathy, as well as the safety profile of these drugs. We identified 13 patients seen between 2000 and 2019, with a median age at presentation of 66 years (range 53–89); 11 were females. At onset of symptoms, patients were on CQ or HCQ for a minimum of 6 months and up to 21 years. Diagnosis was often delayed by a median of 6 months (range 3–48). At presentation, 13 patients reported limb weakness, with five requiring assistance in walking. Ten reported dysphagia, often severe, resulting in marked weight loss or aspiration pneumonia. Nine reported respiratory symptoms, which were multifactorial in four, and four reported severe neck weakness. Myopathy clinical phenotype showed predominant involvement of one or more of the following: proximal limb muscle weakness (12 patients), dysphagia (9), axial weakness (4), and respiratory failure (5). Eleven patients had a cardiac evaluation showing prolonged QT interval in 10 and CQ/HCQ cardiomyopathy (CMP) in four. Ten out of 12 patients markedly improved after discontinuing the medication, but most were left with some residual weakness. Eleven patients had a muscle biopsy showing a myopathy with rimmed vacuoles and marked acid phosphatase reactivity. Nine had elevated creatine kinase level up to 1,199 U/L. Twelve patients had an electromyography (EMG), which showed myopathic motor unit potentials with fibrillation potentials in 11 and myotonic discharges in 3. Higher cumulative dose and longer exposure duration were associated with more severe disability and more common cardiac and swallow involvement, indicating a cumulative dose effect. Herein, we demonstrate that long-term exposure to CQ and HCQ may result in a myopathy with a wide spectrum of clinical presentation and predilection for swallowing, respiratory, and cardiac muscles, often with marked associated morbidity. Once accurately diagnosed and the drug is discontinued, patients usually improve but often fail to return to baseline.

## Introduction

Chloroquine (CQ) and its derivative hydroxychloroquine (HCQ) were originally used as antimalarial drugs. Although their mechanism of action remains not entirely understood, they have affinity to acidic compartments such as lysosomes and inflamed tissue, which results in altering the lysosomal function, autophagy, and signaling pathways including the immune pathways (1, 2). Due to their immunomodulatory effect, they are widely used nowadays in the treatment of connective tissue diseases (CTDs). However, the effect on lysosomal function and membrane stability can affect various tissues including the skeletal muscle. The use of CQ or HCQ can cause a myopathy characterized by the accumulation of autophagic vacuoles, with abnormally increased acid phosphatase (lysosomal enzyme) reactivity, and detection of myeloid or curvilinear bodies on electron microscopy (3, 4). Clinically, CQ/HCQ myopathy has been described as a myopathy predominantly affecting proximal limb muscles, which is usually reversible once correctly diagnosed and the offending drug is discontinued (5–8). There are rare reports of CQ/HCQ myopathy presenting with prominent respiratory failure or dysphagia (9–11).

When a patient with CTD presents with progressive proximal weakness, the possibility of CQ/HCQ myopathy is often overlooked and overshadowed by the consideration of an inflammatory myopathy, steroid myopathy, or simply deconditioning. Therefore, there is a critical need to better define the clinical, laboratory, histopathologic, and electrodiagnostic findings of CQ/HCQ myopathy, in order to expedite diagnosis and improve patient care by defining the extent of vital functions' involvement in this entity. It is also important to determine the safety profile of CQ and HCQ, as well as the optimal dose and duration of treatment, to avoid such complications. In this study, we aimed to address these gaps in knowledge in a large, single-center, well-characterized series of patients with CQ/HCQ myopathy.

## Methods

The Mayo Clinic Institutional Review Board approved the research protocol, and the study was deemed eligible for Health Insurance Portability and Accountability Act (HIPAA) authorization waiver. We performed a search of Mayo Clinic electronic database to identify patients with the diagnosis of CQ or HCQ myopathy seen between 2000 and 2019. Patients were identified by searching for notes where the terms “chloroquine,” “hydroxychloroquine,” or “Plaquenil” were mentioned in the “diagnosis” section of the note along with the term “myopathy.” We then performed a chart review to identify patients with confirmed diagnosis. Patients were included if they had a biopsy-proven CQ/HCQ myopathy, as described in the introduction, or a definite clinical diagnosis. To be diagnosed clinically, there had to be a well-documented association between exposure to the offending drug and the myopathy, and subsequent improvement after discontinuing the drug, with no other potential culprit medication. We reviewed demographic and clinical characteristics of the patients; serological, electrodiagnostic, and muscle biopsy data; and the results of pulmonary function tests, overnight oximetry, electrocardiogram, echocardiogram, and swallow studies. For a muscle group to be included in the clinical phenotype, the patient must report related symptoms and demonstrate weakness on manual motor examination or ancillary testing (such as swallow evaluation or pulmonary function test). For instance, if a patient reports only limb weakness but displays mild neck weakness on examination, “neck weakness” would not be included in the clinical phenotype. Motor examination was reported as a summated motor Neuropathy Impairment Score (m-NIS) (12). The following eight muscle groups were included in the m-NIS: neck flexors, neck extensors, shoulder abductors, elbow flexors, elbow extensors, fingers abductors, hip flexors, knee extensors, and ankle dorsiflexors. The score for individual muscles ranges from 0 (or normal strength) to 4 (completely paralyzed muscle), and subsequently, m-NIS ranges from 0 to 64. The modified Rankin scale (mRS) score was used to measure disability, reflected by the ability to perform activities of daily living as reported on a questionnaire filled by the patients (13). The scale ranges from 0 for no symptoms to 6 or death. Evaluation of the treatment response was based on clinical examination and patient-reported outcomes as well as improvement in laboratory tests, such as creatine kinase (CK) level, swallow evaluation, or transthoracic echocardiogram.

### Cardiac Evaluation

A heart failure cardiologist (OFA) performed individualized chart review to evaluate for cardiac involvement. Patients' demographics, comorbidities, exposures, cardiac biomarkers, electrocardiograms, echocardiograms, cardiac MRI, and clinical course were reviewed. Owing to the retrospective nature of the study and availability of sufficient diagnostic clinical data, patients were categorized as having (a) “definite” (biopsy-proven) CQ/HCQ cardiomyopathy (CMP), (b) “most likely” CQ/HCQ CMP (alternative etiology absent and concordant clinical course), (c) “possible” but low-likelihood CQ/HCQ CMP (alternative etiology present), or (d) no CQ/HCQ CMP. We only considered patients in the definite or most likely categories as having CQ/HCQ CMP.

### Statistical Analysis

Given the low number of patients, only descriptive statistics were performed. Continuous variables were reported as medians and ranges, and categorical variables as percentages.

## Results

### Clinical Characteristics

We identified 13 patients with CQ/HCQ myopathy: two were on CQ, 10 on HCQ, and one on a combination of both. All patients were Caucasian and were using CQ or HCQ to treat underlying CTD. Baseline characteristics are summarized in Table 1. Three patients were on low dose of prednisone (5–15 mg per day).

**Table 1:** Baseline characteristics.

**Demographic data**	
Age at presentation	66 years (53–89)
Sex	11 females (85%)
Time from onset of symptoms till presentation	6 months (3–48)
Height	162.5 cm (150–176)
Weight	61.5 kg (43–104)
**Offending-drug data**	
Medication used, daily dose: n^*^	Chloroquine 500 mg: 3
	Hydroxychloroquine 400 mg: 11
Daily dose per weight^*^	Chloroquine: 8.8 and 11.6 mg/kg Hydroxychloroquine: 5.85 mg/kg (3.85–8.3)
Exposure duration at onset of neurological symptoms	114 months (6–258)
Cumulative dose at onset	1,830 g (73–3,148)
Concomitant use of statins or prednisone: *n*	Statin + prednisone, 2; statin + pantoprazole, 1; statin only, 1; prednisone, 1
Treated condition: *n*	Lupus erythematosus, 5
	Overlapping connective tissue disease, 2
	Seronegative inflammatory arthritis, 2
	Morphea, 1
	Sjögren syndrome, 1
	Scleroderma, 1
	Undifferentiated connective tissue disease, 1

* *One patient was taking both medications and had no documented weight. Categorical variables are presented as percentages, and continuous variables as median (range)*.

#### Patient-Reported Symptoms

Table 2 summarizes the patients' clinical characteristics. Dysphagia was most commonly severe with significant weight loss ranging from 25 to 85 lb, and two patients had aspiration pneumonia. Among the nine patients who reported respiratory symptoms, dyspnea was attributed to non-neuromuscular causes in four: three with CMP and one with interstitial lung disease and history of pulmonary embolism. Patients with neck weakness mostly reported marked difficulty lifting their head up when lying supine and one reported head drop. Nine patients (69%) reported myalgia.

**Table 2:** Clinical characteristics.

**Patient**	**Age at presentation (years)**	**Sex**	**Patient-reported symptoms**							**Examination highlights**			
			**Dysphagia**	**Dysarthria**	**Dyspnea**	**Neck weakness**	**Limb weakness**	**Difficulty with stairs**	**Marked weight loss**	**Myopathy phenotype**	**Gait aid**	**m-NIS**	**PN**
1	58	F	N	N	N	N	Y	Y	N	D	N	2	Y
2	89	M	Y	Y	N	N	Y**-**minimal	N	Y	A^*^	N	4	N
3	66	F	N	N	Y	N	Y	Y	N	D	N	10	N
4	59	F	Y	N	N	N	Y	Y	Y	A+D	N	14	N
5	62	M	Y	N	N	N	Y	Y	N	D	N	2	Y
6	78	F	Y	N	Y	Head drop	Y	Y	Y	A+B+C+D	Y-walker	18	Y
7	53	F	Y	N	Y	N	Y	Y	N	A+C+D	N	18	N
8	63	F	Y	N	Y	N	Y	N	N	A+C+D	Y-walker W/C	6	Y
9	70	F	Y	N	Y	Can't lift head up	Y	Y	Y	A+B+C+D	N	28	Y
10	73	F	N	N	Y	Can't lift head up	Y	Y	N	B+D	Y-W/C	24	N
11	58	F	Y	Y	Y	Can't lift head up	Y	N	Y	A+B+D	N	19	Y
12	68	F	Y	Y	Y	N	Y	Y	N	A+C+D	Y-W/C	24	N
13	69	F	Y	N	Y	N	Y	Y	N	A+D	Y-W/C	11	NA
Total			10/13 (77%)	3/13 (23%)	9/13 (69%)	4/12 (33%)	13/13 (100%)	10/13 (77%)	5/13 (38%)		5/13 (38%)	14 (2–28)	6/12 (50%)

*A, dysphagia; B, neck weakness; C, respiratory failure; D, proximal weakness; N, no; PN, length-dependent peripheral neuropathy; Y, yes; W/C, wheelchair*.

* *Patient had mild horizontal and moderate vertical ophthalmoparesis, mild neck flexion, and shoulder abduction weakness*.

#### Physical Examination

Table 2 shows details of clinical examination. Limb involvement was predominantly proximal affecting both upper and lower limbs, with either normal or mild distal involvement. Axial involvement affected mostly the neck muscles involving the flexors in four and extensors (head drop) in one. Six patients had a superimposed peripheral neuropathy related to diabetes mellitus in two: chemotherapy induced in one and asymptomatic in one. In the remaining two patients, no other cause for the peripheral neuropathy was identified, and symptoms started after initiating CQ/HCQ. However, an association between the neuropathy and the drug was not established with certainty in either case.

### Laboratory Findings

Results of the serum laboratory work up are summarized in Table 3. Elevated CK level was observed in 9/13 (69%) tested patients, and aldolase level in 2/8 (25%). Only four out of 12 patients had elevated creatinine levels with an estimated glomerular filtration rate of <60 ml/min/body surface area (BSA) (range 28–51).

**Table 3:** Cardiac findings.

**Patient**	**Muscle enzymes**		**Cardiac testing**							**Kidney function**	**Respiratory function**
	**Creatine kinase (U/L)^a^**	**Aldolase (U/L)^b^**	**Troponin-T (ng/ml)^c^**	**NTproBNP (pg/ml)^d^**	**QT interval duration (ms)^e^**	**Ejection fraction (%)**	**Increased wall thickness (Y/N)**	**Restrictive vs. dilated CMP**	**CQ/HCQ CMP**	**Creatinine (mg/dl)^f^**	**Vital capacity (%)^g^**
1	110	6.8			**447**	66	N		No	**1.1**	89
2	**557**										
3	**244**	5.1	**0.12**	**2,896**	**457**	72	Y	R	Most likely	**1.2**	89
4	**651**	**8.5**	<0.01		**468**	70	N		No	0.9	123
5	125	6.4			**448**	65	N		No	0.8	82
6	**1,199**	**35.7**	**0.76**	**8,812**	**498**	32	N	D	No	1	**33**
7	149	4.1			**447**	50	N	R	Possible	0.5	**47**
8	**403**				**493**	70	Y	R	Most likely	0.9	**68**
9	**560**		**0.09**	**4,786**	432	49	N	R	No	0.8	**58**
10	**337**	6.4								**1.1**	79
11	**386**		**0.17**	**4,277**	**560**	69	Y	R	Most likely	0.9	
12	135	6.9	**0.13**	**7,255**	**476**	27	N	R	Possible	**2.8**	**61**
13	**302**		**0.36**	**25,681**	**634**	33	Y	R	Definite	**1.1**	

*Abnormal values are in bold*.

Normal range:

a 38–176 U/L, except patients #2 and #5: 52–336 U/L;

b <7.7 U/L;

c <0.01 ng/ml;

d ≤ 206 pg/ml;

e *400–440 ms*.

f *Females: 0.59–1.04 mg/dl and males 0.74–1.35 mg/dl*.

g *≥70% of predicted*.

*CQ, chloroquine; HCQ, hydroxychloroquine; CMP, cardiomyopathy; D, dilated cardiomyopathy; NTproBNP, N-terminal pro-brain natriuretic peptide; R, restrictive cardiomyopathy*.

#### Electrodiagnostic Testing

Nerve conduction studies and needle electromyography were performed in 12 patients. Eleven had abnormal spontaneous activity with fibrillation potentials, while myotonic discharges were present only in three. The fibrillation potentials were usually present diffusely in the axial, proximal, and distal muscles and occasionally in cranial muscles. Six of 12 patients had a superimposed length-dependent, sensory and motor, predominantly axonal peripheral neuropathy. Only one patient had repeat electromyography (EMG) 3 months after discontinuing the drug, which continued to show fibrillation potentials and myopathic motor unit potentials diffusely. At that time, the patient reported improvement in his symptoms without returning to normal.

#### Muscle Biopsy

**Figure 1:**
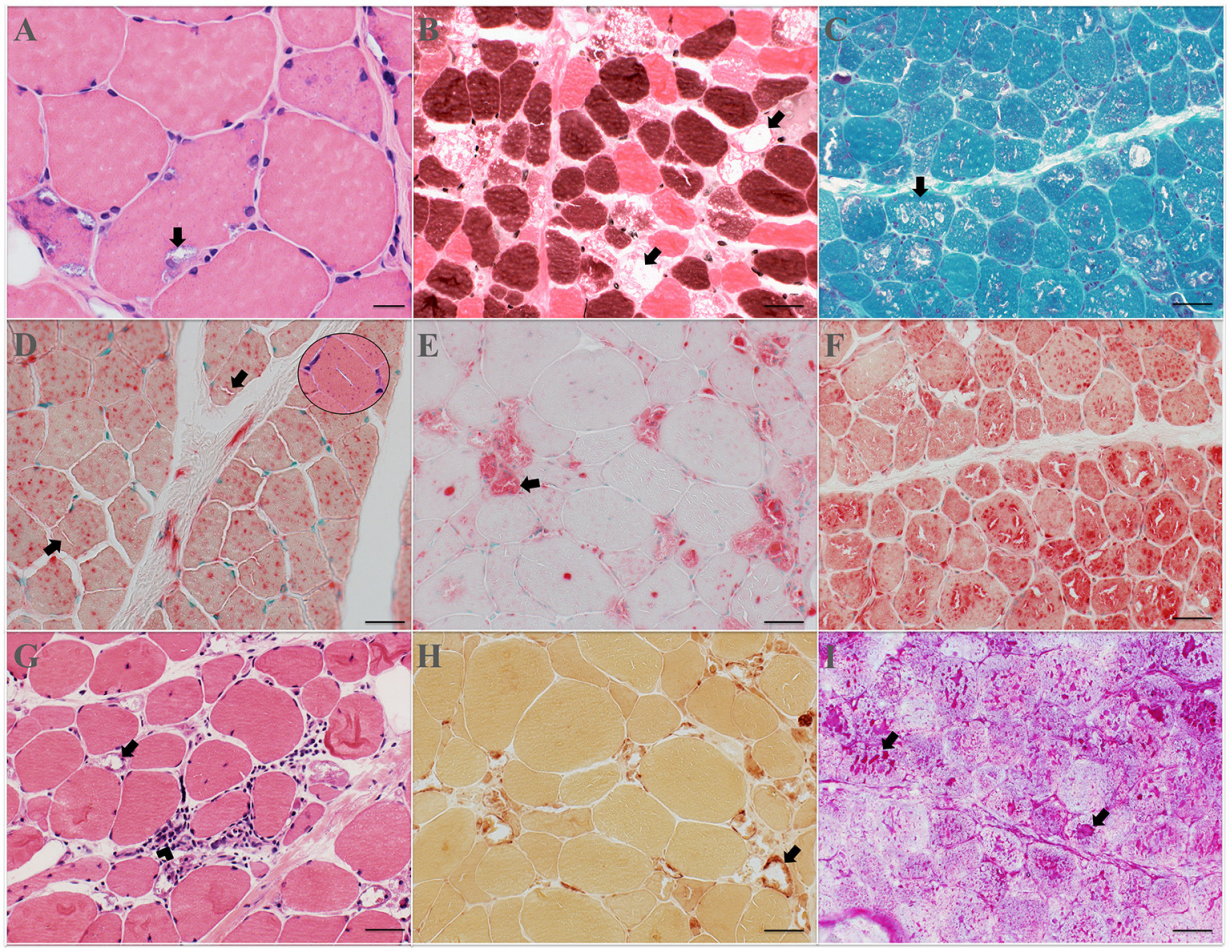
Muscle biopsy findings. Muscle frozen sections stained with hematoxylin and eosin (H&E) **(A,G)**; ATPase at pH 4.3 **(B)**; modified trichrome **(C)**; acid phosphatase **(D–F)**; non-specific esterase **(H)**; and periodic acid Schiff **(I)**. CQ/HCQ myopathy is characterized by the presence of rimmed vacuoles, some of which contain granular material, and increased acid phosphatase reactivity. The vacuoles (arrows) may be of a small size **(A)**, or large occupying the majority of the muscle fiber **(B)**, or fibers may harbor multiple vacuoles of various sizes **(C)**. Rarely, vacuoles may be slit-like **(D)**, also shown in H&E in the top right corner. Vacuoles occur in both type 1 and type 2 fibers **(B)**, but more commonly in type 1. Increased acid phosphatase reactivity may occur in a punctate fashion (“measles” appearance) **(D)**, within vacuoles **(E)**, or both **(F)**. Occasionally, a mild inflammatory reaction may be present (arrowhead) either in the endomysium **(G)** or perimysium. Some of the vacuoles may display increased non-specific esterase reactivity **(H)**, or glycogen accumulation **(I)**. Scale bar: **(A)**, 20 μm; **(B–I)**, 50 μm. CQ, chloroquine; HCQ, hydroxychloroquine.

Eleven patients underwent a muscle biopsy: five from the deltoid, two from the quadriceps, one from the biceps, one from the triceps, one from the tibialis anterior, and one from the endomyocardium. Endomyocardial biopsy findings have been previously published, showing marked sarcoplasmic vacuolization with myeloid and curvilinear bodies on electron microscopy (14). The findings on the skeletal muscle biopsies are highlighted in Figure 1. All biopsies demonstrated a myopathy with rimmed vacuoles and marked acid phosphatase reactivity, except in patient #2 in whom vacuoles were exceedingly rare; but most muscle fibers showed a marked increase of punctate acid phosphatase reactive deposits. Half of the specimens displayed a very mild inflammatory reaction, mainly perivascular, except for patient #6, who had small to moderate collections of inflammatory cells at a few perivascular sites. Occasional necrotic and necrotic fibers were seen in 7/10 specimens. Mitochondrial abnormalities, when present, were mild and likely age appropriate, except in patient #8 who had about one or two cytochrome *c* oxidase negative fibers per fascicle. Mildly increased endomysial connective tissue (fibrosis) was only seen in patient #4.

#### Cardiac Testing

Eleven patients had sufficient clinical data available to evaluate for cardiac involvement. Results are shown in Table 3. Of those, one had “definite” biopsy-proven CQ/HCQ CMP, and three were deemed to “most likely” have a CQ/HCQ CMP. Two patients had a “possible” but low-likelihood CQ/HCQ CMP, as one had concomitant exposure to doxorubicin and one had underlying tachycardia-mediated CMP with underlying tachycardia-mediated CMP. The patient with a “definite” CQ/HCQ CMP diagnosis had the highest levels of biomarkers of myocardial necrosis (troponin-T) and increased wall strain [N-terminal pro-brain natriuretic peptide (NTproBNP)].

#### Respiratory Testing

Ten patients underwent pulmonary function test. It showed a restrictive pattern, consisting of reduced vital capacity (VC) with preserved FEV1 (forced expiratory volume in 1 s)/forced VC ratio in five patients. VC results are shown in Table 3. Only three of 10 patients had maximal respiratory pressures measured, and these were reduced in two. Overnight oximetry showed sleep-related disordered breathing in four out of four tested patients.

#### Barium Swallow

Only four patients had a barium swallow evaluation, which was abnormal in all four. Patients #2 and #6 had severe pharyngeal dysphagia with airway penetration and aspiration. Patient #2 had additionally a cricopharyngeal bar with significant retention. Patients #9 and #12 demonstrated a milder degree of oropharyngeal dysphagia with no aspiration at presentation to our institution; both were reported to have more significant swallowing difficulty upon prior evaluation locally. In regard to the esophageal phase of swallowing, patient #9 had a cervical esophageal web and patient #13 diminished esophageal peristalsis and numerous tertiary contractions. Patient #5 had longstanding esophageal dysphagia due to dysmotility and strictures attributed to her scleroderma; and hence, dysphagia was not included in her myopathy clinical phenotype.

### Correlation of Clinical Findings With Drug Exposure Duration and Cumulative Dose

**Figure 2:**
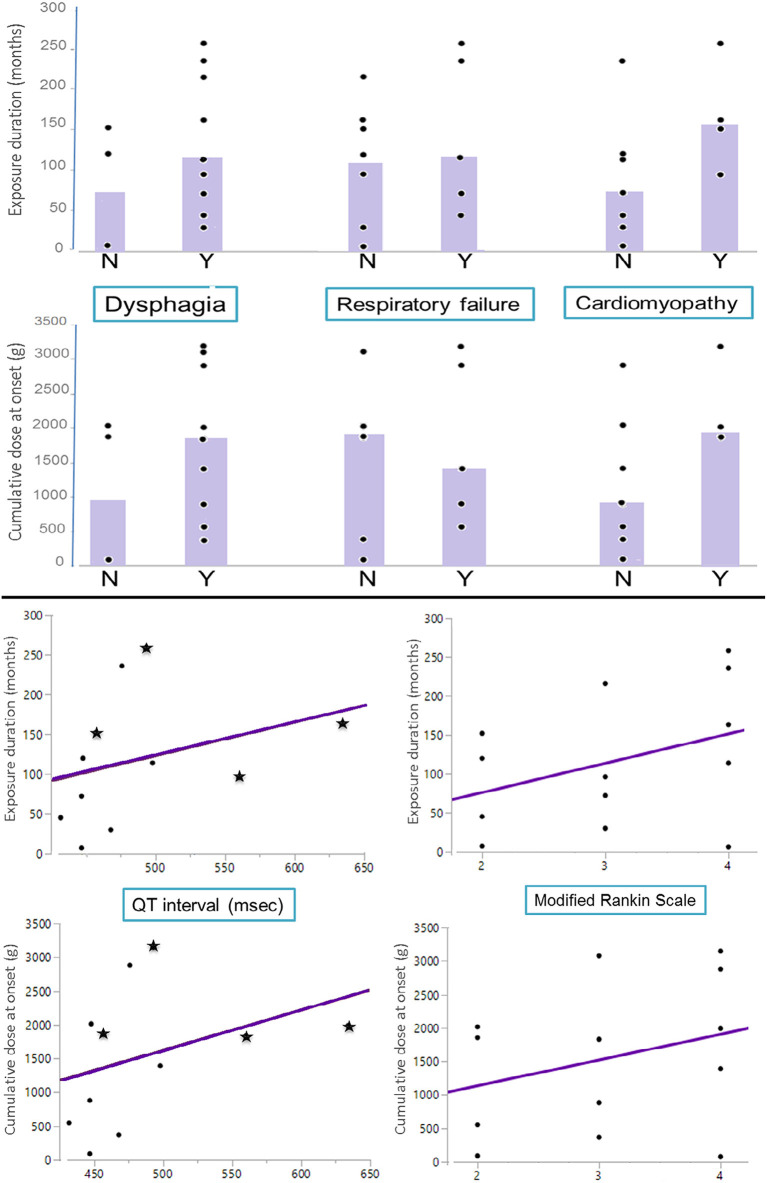
Correlation of clinical findings with drug exposure duration and cumulative dose. **(Top)** Exposure duration and cumulative dose at onset of neurological symptoms (*y*-axis) in patients with or without dysphagia, respiratory failure, or cardiomyopathy (*x*-axis). Bar graphs represent the median value in each group. **(Bottom)** Scatter plot of exposure duration and cumulative dose at onset of neurological symptoms (*y*-axis) by QT interval and modified Rankin scale (*x*-axis) with line of best fit. In the QT interval graphs, patients with associated chloroquine/hydroxychloroquine cardiomyopathy are displayed as “stars”.

Patients with higher cumulative doses and longer exposures had more severe disability as reflected by their mRS and more common swallowing and cardiac muscle involvement. Patients with CQ/HCQ cardiomyopathy had longer QT duration than patients without: median of 525 ms (457–634) vs. 458 ms (432–498), respectively. Detailed results are shown in Figure 2.

### Treatment Outcomes

Clinical follow-up was available in all patients except patient #8. Patient #6 decided to go with hospice care due to all her comorbidities and passed away 2 months after her presentation. The remaining 11 patients reported significant improvement soon after stopping the offending drug. Limb and neck weakness markedly improved but never back to normal except in one patient who had only mild weakness at presentation (patient #1). Patients mostly demonstrated residual proximal lower limb weakness. Similarly, dysphagia and dyspnea improved as well, with some patients reporting complete resolution of their symptoms. Patient #2, who was on dual therapy, stopped CQ first and reported, 6 months later, only mild improvement of his dysphagia but significant improvement of his limb weakness (which was mild) and normalization of his CK level. HCQ was then stopped with marked improvement of his dysphagia demonstrated by a repeat swallow evaluation. Among the nine patients with elevated CK level at diagnosis, four had their CK level rechecked after discontinuing the medication, and it returned to normal in all patients. It is noteworthy that stopping CQ or HCQ had consequences in four patients: flare-up of severe arthralgia in three, one of whom was subsequently diagnosed with (newly) seropositive rheumatoid arthritis, and alopecia flare-up in a patient with systemic lupus erythematosus.

## Discussion

The clinical phenotype associated with CQ/HCQ myopathy has been mainly described in the literature as a proximal myopathy (5, 8, 15). In this study, we demonstrate that CQ/HCQ myopathy is associated with a wide spectrum of clinical presentations consisting of one or a combination of the following: proximal limb, swallowing, respiratory, and axial muscle weakness. The predilection for muscles associated with vital functions, in addition to common cardiac involvement, may result in marked morbidity and potential mortality if not diagnosed accurately and timely. The marked swallowing involvement has not been highlighted in the literature, and as shown here, it can result in significant weight loss, frailty, and aspiration pneumonia. Dysphagia may be the main predominant feature of CQ/HCQ myopathy as seen in our patient (#2) who has been previously reported, or in combination with severe neck and shoulder weakness as in patient #11, whose chief complaint was inability to lift his head off the pillow (10, 16). This brachio-cervical phenotype can be mistaken for a CTD-associated inflammatory myopathy (17). The only patient who presented with a head drop rather than severe neck flexion weakness had also a mild inflammatory component on her muscle biopsy, although the predominant finding was that of a myopathy with rimmed vacuoles. So it remains unclear if this contributed to the novel CQ/HCQ myopathy phenotype, as myopathic head drop can be seen in inflammatory myopathies as well (18). Lastly, CQ/HCQ myopathy can be associated with significant neuromuscular respiratory insufficiency, as previously reported (9, 10). Herein, we show that neuromuscular respiratory insufficiency is common and should be screened for routinely in this patient population.

Regarding other neuromuscular complications of CQ and HCQ, peripheral neuropathy is usually seen in association with a myopathy, and there are rare case reports of CQ/HCQ-related myasthenia gravis (19, 20). This is in keeping with findings in animal models, where muscle fibers were much more severely affected than peripheral nerves and neuromuscular junction (3). Furthermore, in most previously reported cases of CQ/HCQ neuropathy, a myopathy was indeed the predominant clinical findings manifesting with progressive proximal weakness (21–23). It is noteworthy that given the common predilection for bulbar and neck muscles, CQ/HCQ myopathy could be mistaken for myasthenia gravis. Per previous reports, patients may have an overlap with predominantly a myopathy and a mild neuromuscular transmission defect component, or CQ/HCQ may exacerbate underlying autoimmune myasthenia gravis (24, 25).

When present, abnormal spontaneous activity on EMG or an elevated CK level would strongly argue against a corticosteroid-induced myopathy or deconditioning. However, the clinical and electrodiagnostic features or CQ/HCQ myopathy are indistinguishable from an inflammatory myopathy. Therefore, a muscle biopsy is often needed to establish a diagnosis. It is crucial to include a clinically involved muscle, as sampling error may happen with both entities. While it would be difficult to miss the diagnosis in cases with prominent large vacuoles, the histopathologic findings may be more subtle in some cases as shown here. Furthermore, there may be minimal associated inflammation that could mislead interpretation. The classic finding of myeloid or curvilinear bodies is seen on electron microscopy, which may not be universally available and has inherent technical challenges (3, 4).

Similar to previous reports, all patients improved after the offending drug was stopped. However, the CQ/HCQ myopathy often resulted in significant morbidity and impact on the patient's life. This is in part due to the delay in diagnosis by a median of 6 months and up to 4 years and the pleiotropic effect of the drugs with predilection for vital organs. Once diagnosed, the patients do improve but often are left with residual weakness. In addition to accurately diagnosing patients, it is crucial to recognize and screen for swallowing, as well as respiratory and cardiac involvement, all of which were common in our series. This would allow diet modification to avoid aspiration pneumonia, appropriate nutrition supplementation to fight weight loss, administering non-invasive ventilator support for the sleep-related disordered breathing, and monitoring for cardiomyopathy and prolonged QT intervals that predispose to lethal cardiac arrhythmias and complications, with echocardiography and electrocardiograms, respectively (26, 27).

There is a major gap in understanding the mechanism of action of CQ and HCQ, their safety profile, and recommended dosing (15). Herein, we showed that longer exposure and higher cumulative doses are associated with higher disability and more common swallowing and cardiac involvement. Neuromuscular complications seem to be a late complication, as none of our patients and only very rare cases reported by others developed a myopathy with <6 months of exposure to CQ or HCQ (6, 15). Hence, it would be less likely for a brief exposure to these drugs, such as when used during a viral infection, to cause a myopathy. Nevertheless, CQ/HCQ myopathy could theoretically occur in this context when taking the drug for a prolonged period of time as a preventive measure. This is similar for retinal toxicity, as the risk of developing it with <5 years of exposure is <1% (28). However, it is important to highlight that this is not the case with the potentially-lethal cardiac arrhythmias, mainly torsade de pointes, which may occur even after brief exposure to these drugs (26). All of our patients, except one, had evidence of prolonged QT interval. This could potentially be explained by the difference in underlying mechanisms. Injury of skeletal or cardiac muscle and retinal tissues is related to the drug interfering with lysosomal function and stability of cell membranes, with subsequent accumulation of waste products over time that eventually result in clinical manifestations, often affecting multiple organs or tissues. On the other hand, cardiac arrhythmias are due to blocking of *KCNH2-*encoded hERG/Kv11.1 potassium channel and prolonging the QT interval (26). Further studies are needed to better elucidate the mechanism of action of HCQ and CQ and to determine underlying risk factors that predispose certain patients to develop complications from these drugs. Pharmacogenomics could be considered especially for cardiac complications, although the rarity of the neuromuscular complications in particular may limit feasibility. It is also noteworthy that CQ/HCQ myopathy is also perhaps under-recognized, as witnessed by the delay in diagnosis with time from symptom onset to presentation to our institution ranging from 3 up to 48 months. This is in part because muscle weakness in patients with CTD is more commonly due to an inflammatory myopathy, steroid myopathy, or deconditioning. Weakness may also be multifactorial; therefore, a contribution of these other risk factors to the clinical phenotypes observed in our patients cannot be excluded. Other limitations of our study include the retrospective study design, sample size, referral bias inherent to a tertiary care center, and lack of a uniform diagnostic approach.

## Conclusion

Herein, we demonstrate that long-term exposure to CQ and HCQ may result in a myopathy with a wide spectrum of clinical presentation and predilection for swallowing, respiratory, and cardiac muscles, often with marked associated morbidity. Once accurately diagnosed and the drug is discontinued, patients usually improve but often fail to return to baseline. The continuous need for CQ or HCQ treatment, and the optimal dosage and treatment duration should be evaluated on a routine basis, along with monitoring of the QT interval, to avoid short-term and long-term complications of these drugs.

## Data Availability Statement

The original contributions presented in the study are included in the article/supplementary material, further inquiries can be directed to the corresponding author/s.

## Ethics Statement

The studies involving human participants were reviewed and approved by Mayo Clinic Institutional Review Board. Written informed consent for participation was not required for this study in accordance with the national legislation and the institutional requirements.

## Author Contributions

EN, PP, and OA contributed to the study concept and design, acquisition, analysis, interpretation of data, drafting of the manuscript, and critical revision of the manuscript for important intellectual content. EN prepared the first draft of the manuscript and supervised the study. All authors contributed to the article and approved the submitted version.

## Conflict of Interest

The authors declare that the research was conducted in the absence of any commercial or financial relationships that could be construed as a potential conflict of interest.
